# Evaluating the Distinction between Cool and Hot Executive Function during Childhood

**DOI:** 10.3390/brainsci13020313

**Published:** 2023-02-13

**Authors:** Yusuke Moriguchi, Steven Phillips

**Affiliations:** 1 Graduate School of Letters, Kyoto University, Yoshidahoncho, Kyoto 606-8501, Japan; 2 Mathematical Neuroscience Group, Human Informatics and Interaction Research Group, National Institute of Advanced Industrial Science and Technology (AIST), Tsukuba 305-8566, Japan

**Keywords:** executive function, cool–hot, the prefrontal cortex, children, development

## Abstract

This article assesses the cool–hot executive function (EF) framework during childhood. First, conceptual analyses suggest that cool EF (cEF) is generally distinguished from hot EF (hEF). Second, both EFs can be loaded into different factors using confirmatory factor analyses. Third, the cognitive complexity of EF is similar across cEF tasks, and the cognitive complexity of cEF is similar to hEF tasks. Finally, neuroimaging analysis suggests that children activate the lateral prefrontal regions during all EF tasks. Taken together, we propose that the cool–hot framework is a useful, though not definitive way of characterizing differences in EF.

## 1. Introduction

Executive function (EF) refers to an ability to control thought and emotion [1,2]. This cognitive ability first develops during infancy, then undergoes rapid changes during childhood [3,4]. Several previous studies have reported the important role played by the EF in multiple child development areas. For example, EF skills are associated with cognitive development (e.g., theory of mind) and academic achievement [5,6,7,8]. Thus, researchers have focused on improving children’s EF skills using training or educational programs [9,10,11].

The present study aimed to use theoretical, behavioral, and neuroimaging perspectives to assess a conceptual framework, the cool–hot EF framework, during childhood; in this article, it is proposed that, though the framework was useful, it was not decisive for characterizing differences in EF. Extant relevant literature has provided several models for characterizing children’s EF. One such influential model was proposed by Miyake et al. [12]; it incorporates inhibitory control, cognitive shifting, and working memory when using confirmatory factor analyses. Inhibitory control is the cognitive ability that governs the suppression of a dominant response; it is assessed using Stroop tasks and go/no-go tasks. Cognitive shifting is the cognitive ability that governs flexible switching between different tasks or mental sets. This ability is often assessed using task-switching paradigms. Finally, backward digit span tasks or visuospatial tasks are often used for indexing working memory (or updating), which enables online maintenance and manipulation of information. A subsequent study reported that, rather than the three-component model described above, the components of EF can be deconstructed into one EF factor that is common across all EF tasks and some specific factors that are unique to a particular task (i.e., shifting and updating) [13]. In developmental studies, the EF factor structure can differentiate over the course of child development. That is to say, one general factor can adequately explain preschool children’s performances in EF tasks, while two- or three-factor models are appropriate for explaining school-aged children’s or adolescents’ performance in EF tasks [14,15,16].

The EF skills mentioned above are often classified as “cool” EF (cEF) skills. A framework of cool and hot distinctions in EF has been proposed [17,18]. Under the framework, cEF refers to cognitive skills that work in neutral, abstract, and decontextualized problem situations (e.g., Stroop tasks). Recent studies have also focused on more affective aspects of EF such as hot EF (hEF); the term hEF describes processes that have been elicited under affective conditions and such tasks consist of motivationally salient situations (e.g., seeking and regulating the impulse to achieve rewards). hEF also involves deliberate emotion regulation, which modulates approach–avoidance reactions. In a delay-of-gratification task, for instance, children are required to inhibit their impulse to eat a single marshmallow that has been placed in front of them in exchange for receiving two marshmallows later [19].

Several studies have used the cool–hot framework to examine EF development during childhood. For example, researchers have examined whether cool and hot EFs tend to develop similarly or differently [20,21]. Past research has shown that the developmental trajectory of cool and hot EFs may be similar during childhood and that they start to differ during adolescence; while hEF shows somewhat nonlinear changes, cEF exhibits linear changes [17,22]. Furthermore, in applied and educational contexts, cool and hot EFs may be differently related to real-world school behaviors. Indeed, several studies have reported that hEF is strongly associated with behavioral problems, whereas cEF is more related to academic achievement (e.g., math) [23,24,25]. Moreover, in children with atypical development, the cool and hot distinction is useful for characterizing comorbidities across diagnostic categories and the heterogeneities within them [26].

However, research has not always supported the different roles of cool and hot EFs [27], suggesting that the cool–hot distinction may not be as decisive as previously thought. Indeed, Zelazo and Carlson [17] noted that both aspects of EF can work together and may not be clearly separated. Moreover, Welsh and Peterson [21] indicated that the cool–hot framework poses some challenges in terms of its components’ classification and construct validity.

Based on these proposals, we assessed the cool–hot EF framework during childhood. In this paper, we first address the issues around cool and hot EF classification and the framework’s construct validity. Moreover, we also explore the cognitive complexity and neural basis of the cool and hot EFs.

## 2. Classifications of Cool and Hot EF Tasks

Several tasks have been used for assessing cool and hot EFs, but the classifications of some of these tasks have differed across studies. As suggested by Welsh and Peterson, one possible solution for classifying cool and hot EFs involves considering “the temperature” of a given EF task (e.g., reward salience). Moreover, hEF involves modulating approach–avoidance reactions, which represents a distinction from cEF [28]. In our current study, we classified EF tasks conducted during early childhood as shown in Table 1.

First, it should be noted that tasks assessing cognitive skills in neutral situations that do not include clear motivational stimuli (rewards) can be regarded as cEF tasks. For example, Dimensional Change Card Sort (DCCS) is a widely used cEF task among young children [29,30]. In the DCCS task, children are instructed to sort cards with two dimensions (e.g., shape and color—yellow cups, yellow stars, green cups, green stars and so on). In the preswitch trials, children are instructed to sort cards based on one dimension (e.g., color). After performing the preswitch phase, the children are instructed to sort the cards based on the other dimension (e.g., shape) during the postswitch phases. Several studies reported that 3-year-old children find it difficult to switch the rules and so continue sorting the cards based on the first rule, while 4- and 5-year-old children perform this task correctly.

Another task involving cEF is the Stroop-like task [31,32]. In a day/night task, children are instructed to say “day” when given a moon card and say “night” when given a sun card. Children have a natural tendency to associate “day” with a sun card and “night” with a moon card; therefore, children participating in this task must inhibit this tendency to respond in accordance with the instructions. In this task, younger children failed to inhibit their natural tendency, whereas 5-year-old children performed the task correctly.

There are some other cEF tasks, as well. In the grass/snow task, children need to respond to the white card when the experimenters say “grass” and respond to the green card when they say “snow”. In the bear/dragon task, children need to obey the bear’s instructions (such as touch your nose), but disobey the dragon’s. In Luria’s hand game, children are told to produce a motion that is the opposite of what the experimenter does (e.g., children must point a finger when the experimenter makes a fist).

However, some variants of cEF tasks can include “temperature”. For example, let us consider the emotional face version of the DCCS task. This version involves facial stimuli with emotional expressions (e.g., happy, sad, or neutral) instead of the shapes and colors of the standard DCCS task [33]. Results have revealed that children tend to perform better on the emotional face version of the DCCS task rather than the standard one; this suggests that the emotional aspects included in the stimuli of the emotional face version may affect children’s EF. Furthermore, other studies have shown that children’s performances of cEF tasks may be affected by rewards. Tarullo et al. [34] reported that, in the DCCS task, during postswitch phases with rewards, children showed greater accuracy with slower reaction times compared to those without rewards. Similar effects have been observed in research involving Stroop-like tasks [35]. Thus, there may be some hot aspects even in traditional cEF tasks.

Second, some tasks that include clear motivational stimuli (rewards) and modulating approach–avoidance reactions can be regarded as hEF. A children’s version of a gambling task is widely used for assessing hEF [36]. Children are required to choose between two options. One option includes cards offering more candies per trial but occasional large losses across trials (a disadvantageous option). The other included cards offering fewer rewards per trial and fewer losses across trials (an advantageous option). The results show that 4-year-old children tend to choose advantageous options compared to 3-year-olds.

Moreover, delay-of-gratification tasks are also used for assessing hEF. In such tasks, the ability to resist accepting a smaller immediate reward in favor of a larger delayed reward is tested. The “marshmallow test”, where children must select between two options—eating one marshmallow right now or waiting for a certain amount of time in exchange for two marshmallows—is a traditional method for evaluating children’s ability to delay gratification [19]. Similarly, in another version, children must select between a smaller immediate reward (one sticker now) or a larger delayed reward (five stickers after the experiment) [37]. Both have found that, compared to younger children, older children tend to select the delayed option.

One further “cool–hot” assessment framework can be found within the delay-of-gratification literature. Metcalfe and Mischel [38] proposed that while cool processes influence the hot processes and, therefore, require complicated cognitive and conscious thinking, hot processes are associated with impulsive emotional processing and response. It should be noted that this framework differs from the cool–hot EF framework because, compared to hEF, the hot processes in the Metcalfe and Mischel framework do not include regulation processes [17].

In some variants of hEF tasks, the reward values may be reduced. Prencipe and Zelazo [39] gave young children two versions of a delay-of-gratification task. In one version, the children were given a smaller immediate option and a larger delayed option and then asked to choose their desired rewards. This normal version is regarded as hot. The other version provided the children with two options and asked them which reward the experimenter would choose. In this version, the children did not receive any rewards, meaning this version could be regarded as cold. The results showed that children were more likely to choose a larger delayed reward in the cool version compared to the hot version; this suggests that the reward values had been reduced in the cool version.

Third, yet another type of task can include both aspects of EF. The task is a less-is-more task [40]. Children are given two options in this task, one of which has a smaller reward and the other a larger reward; they must choose the smaller reward if they want the larger reward. As a result of the possibility of getting rewards and the potential need to control impulsivity and motivation, this task involves hEF. In addition, given the children’s need to control their tendency to choose an option with larger rewards, this task also utilizes cEF.

We have now reviewed several tasks that include both cool and hot EF aspects. Strictly speaking, cEF tasks with rewards (e.g., a DCCS task with sticker rewards) can be distinguished from hEF tasks. In hEF tasks, children must regulate their impulse to select a reward and modulate approach–avoidance reactions, whereas in the cEF tasks with rewards, they must regulate their behavior in order to achieve rewards. Furthermore, hEF tasks that have decreased reward values cannot be regarded as cEF tasks [39] because such tasks (e.g., a modified cool version of the delay-of-gratification task) do not always involve cognitive processes such as inhibitory control and cognitive shifting. Here, we would like to suggest that cEF cannot be clearly distinguished from hEF simply based on the inclusion of clear motivational stimuli.

## 3. Factor Structure

Next, we examined the construct validity of two aspects of EF. Previous research examined whether cool and hot EFs can be separable during early childhood. In a classic study, Hongwanishkul et al. [20] provided children with a working memory task and a DCCS task as cEF tasks, and a gambling task and a delay-of-gratification task as hEF tasks and analyzed the developmental patterns of the EF tasks. In the results, the developmental patterns of the cool and hot EF tasks were generally similar. Nevertheless, there were some differences between cool and hot EF in that cEF, but not hEF, was correlated with intellectual functioning and temperament.

However, the correlational approach may not be sufficient for assessing whether cool and hot EF are separable. Factor analysis is one useful approach in this regard. As noted above, previous research reported that one general factor is sufficient for explaining children’s performances in cEF tasks. Therefore, the important question here is whether both aspects of EF tasks can be separated or whether they should be loaded into a single factor.

Several studies have reported that a two-factor model, consisting of hot and cool EF factors, provides a better fit compared to a one-factor model [23,25,41,42,43,44,45]. For example, Carlson et al. [43] provided preschool children with a bear/dragon task, a less-is-more task, a grass/snow task, a DCCS task, a working memory task, a gift delay task, a delay-of-gratification task, and a tower task (taking turns to build a tower); they then analyzed the resulting task data using confirmatory factor analysis. Their analysis showed that the bear/dragon task, the less-is-more task, the grass/snow task, the DCCS task, and the backward digit span task loaded as cool (or conflict) EF, while the gift delay task, the delay-of-gratification task, and the tower task loaded as hot (or delay) EF. Montroy et al. [45] also reported that a DCCS task and two types of bear/dragon tasks as well as some other tasks that did not include rewards were loaded as cEF, whereas two delay tasks and teacher-reported EF assessed using the Child Behavior Questionnaire (CBQ) were loaded as hEF tasks. Moreover, a recent study reported that cool and hot EF tasks can be loaded as different factors for a 2-year-old [46].

Although cool and hot EFs can be separated, other research showed that a one-factor model provides a better fit compared to a two-factor model [47,48,49]. Specifically, Allan and Lonigan [47] provided children with a grass/snow task, a task that was similar to a bear/dragon task, and other cEF tasks that did not include rewards; the children also participated in two delay-of-gratification tasks, a less-is-more task, and a box search task. The box search task is a variant of the go/no-go task, where it includes sticker rewards. Allan and Lonigan [48] administered a day/night task, a bear/dragon task, and a task similar to Luria’s tapping task as cEF tasks; furthermore, they provide a “hot” head-to-toes task with rewards, a “hot” grass/snow task with rewards, and a “hot” shape task, which is similar to a Stroop task but includes rewards. The analysis revealed that a one-factor model was most suitable for describing performance in EF tasks.

We noted some differences, in terms of the tasks included and the sample population selected (e.g., high-risk group), between research that reported using a two-factor model and that reported using a one-factor model. Specifically, the research that reported using a two-factor model used completely different tasks for cool and hot EF assessments, but the research that reported using a one-factor model often included similar tasks for cool and hot EF assessments. For example, the box search task included in Allan and Lonigan’s study [47] was originally regarded as an inhibitory control task (cEF task). Similarly, Allan and Lonigan [48] used a “hot” head-to-toes task, a “hot” grass/snow task, and a “hot” shape task, which were also originally regarded as cEF tasks. Allan and Lonigan [48] distributed rewards (points) dependent on the children’s performance, meaning these tasks were regarded as “hot” EF tasks. However, as noted above, such cEF tasks that entailed rewards can be differentiated from original hEF tasks (e.g., delay-of-gratification and gambling tasks). Thus, widespread research seems to support the use of two-factor models over one-factor models, but differentiation between cool and hot EF may depend on the tasks included in factor model studies.

Moreover, the classification of the “less-is-more” task differed across various studies. Here, we surmise that the less-is-more task includes both cool and hot EF because children have to regulate their motivation and impulsivity in order to earn the reward, while they must inhibit their tendency to select a choice that entails a larger reward. However, in research using confirmatory factor analysis, this task was regarded as cEF in one study [43] and general EF in another study [47]. In general, the less-is-more task was more likely to be related to cEF than hEF in previous research.

These results suggest that, although cool and hot EFs can be separated during early childhood, this differentiation may depend on the research study and the selected tasks.

## 4. Cognitive Complexity

Next, we analyze the cognitive complexity of cool and hot EF tasks (Table 2). Specifically, from a cognitive complexity perspective, we propose that there are certain similarities and differences between cool and hot EF tasks. Two theories can explain EF development in terms of cognitive complexity: the cognitive complexity and control (CCC) theory [29,30,50,51], and the relational complexity (RC) theory [52,53].

The CCC theory holds that complexity is a function of the number of levels of embedding inherent in a rule system [54]. The DCCS task includes two incompatible pairs of rules and a higher-order rule that integrates the two rules. Some 3-year-old children can understand simple rules that map conditional relations from different antecedents to consequences, such as “if yellow goes here, then green goes there”. Those relations are mapped by a set of conditions (a higher-order rule), which correspond to a certain color or shape, for example, “if this is the color game, yellow goes here, and red goes there”. Three-year-old children who fail to understand the higher-order rule will continue to use the first rule, even though they need to use the second rule. Older children are usually able to understand the higher-order rule and, therefore, perform the DCCS task correctly.

The RC theory describes “complexity” as the number of task variables that must be related to make a single decision. This framework holds that tasks involving cognitive processes relating to triples of objects are more difficult than tasks involving cognitive processes relating to pairs of objects [53]. A binary (ternary) relation is a subset of pairs (triples) of elements that can be drawn from two (three) sets of elements. Each set can be regarded as one dimension of a variation, or a variable, or an argument that is instantiated by the elements partaking in that relation. Then, relational complexity can be described as the number of such dimensions, variables, or arguments. For example, the statement “A is greater than B” is interpreted as a binary relation that includes two dimensions of variation, or two variable arguments (A and B), where the first dimension is instantiated by the elements in set A, which are larger than some elements in set B, while the second dimension is instantiated by the elements in B, which are smaller than some elements in A.

The RC theory holds that the DCCS task has three arguments: the setting condition (color or shape game), an antecedent condition that assigns attributes (color or shape), and piles [52]. Young children may not have the necessary information capacity for processing these three arguments and may, therefore, need to decompose the task into simpler subtasks (i.e., the complexity is reduced from a ternary relationship into a binary relationship). However, dimensions in the DCCS task are related and conflict with each other, and children cannot reduce their complexity. Indeed, when the task is modified so that the complexity is reduced to binary relations, even young children perform the task correctly.

The CCC theory holds that the task structures of Stroop-like tasks are similar to those of the DCCS task [51]. In a Stroop-like task, simple rules correspond to conditional relations from different antecedents to consequences: for example, “If it is a sun card, then say ‘night,’ and if it is a moon card, then say ‘day.’” Simple rules can be embedded under a higher-order rule in an unusual Stroop task, for example, “If it is the unusual task, then it is a sun card, so then say ‘night.’” The framework of the RC theory provides two arguments: an antecedent condition that assigns attributes (sun or moon) and children’s responses (day or night). However, it is unclear whether the setting condition (unusual task) is an augment. If the unusual game setting remains the same across all trials, then this condition can be regarded as a constant in the Stroop-like task. Alternatively, if there is a usual game condition (e.g., “If it is a sun card, then say ‘day.’”), then the game condition can be regarded as an additional variable—that is to say, there are three arguments in this Stroop-like task. The structures of other cEF tasks, such as grass/snow, bear/dragon, and Luria’s task, are similar to that of the day/night task. Thus, the CCC and RC theories confirm that these cool tasks are all similar to DCCS and Stoop-like tasks in terms of cognitive complexity.

A few studies have examined the cognitive complexity of hEF tasks. Kerr and Zelazo [36] suggested that a children’s version of a gambling task is similar to the DCCS in terms of cognitive complexity. According to Kerr and Zelazo, 3-year-olds can learn how to initially discriminate between the options (e.g., one option has more rewards, and the other option has fewer rewards), but they may not incorporate losses into their initial discrimination. Meanwhile, 4-year-olds may understand a higher-order rule that can coordinate the initial discrimination with any emerging evidence of loss (e.g., one option has more rewards but larger losses, and the other option has fewer rewards but smaller losses).

According to the RC theory, integrating the differences in gains and losses between the decks is necessary to perform the gambling task. In brief, children must think about a ternary relation with three variables (deck, magnitude of gain, and magnitude of loss) for the task. Bunch et al. [55] developed binary and ternary versions of the gambling task. The ternary version was the same as that of Kerr and Zelazo [36], whereas the binary version had decks that varied according to either gains or losses, with the other variable remaining fixed. For example, in the binary version, a gain of one reward and a loss of zero/one reward were offered by one deck. The other deck offered a two-reward gain and a zero-/one-reward loss. In this case, the magnitude of the loss remained the same across options, and the children, therefore, had to consider a binary relation (deck, magnitude of gain). The results showed that even 3-year-olds could choose the advantageous deck in the binary version, but only 5-year-olds were able to choose it in the ternary version.

Finally, using the delay-of-gratification task, the RC theory can explain how there are ternary relations such that the magnitude of one choice (one marshmallow), magnitude of the other choice (two marshmallows), delay, and cognitive complexity in the task are similar to those in the children’s gambling task [56]. While some research shows that the delay-of-gratification task does not have an obvious hierarchical structure, and delay-of-gratification is regarded as being less complex in the CCC theory framework [57], it is also possible that the delay-of-gratification task has a hierarchical structure. There are two rule sets that are in conflict in the delay-of-gratification task. One rule set can concern choosing the options in terms of now/later, and the other rule set can concern choosing them in terms of more/less. Normally, now/more is associated with approach and later/less is associated with avoidance. However, when now is associated with less and later is associated with more, children may need to actively use a higher-order rule to decide based on the more/less rules, rather than the now/later rules.

It should be noted that cognitive complexity does not fully explain children’s performances on EF tasks. For example, studies on the DCCS task have put forward several other theories focusing on attention, graded representation, and negative priming, which may explain why children find it difficult to switch between rules during the DCCS task [58,59,60]. In short, theories focusing on cognitive complexity (the CCC and RC theories) may be unable to fully explain the existing data on outcomes in the DCCS task. Indeed, the cEF discussed in this article may share the same structure in CCC theory and RC theory, but children performed differently across tasks [61]. Several possible factors that can affect performance, such as attentional demand, familiarity, and response type (e.g., card sorting, pointing, or body movement), must be considered. As such, both cognitive complexity and other cognitive processes should be considered when drawing up explanations of children’s performance on EF tasks.

In summary, standard cool and hot EF tasks are considered to have similar levels of cognitive complexity according to the CCC and RC theories.

## 5. Neuroimaging Evidence

The neural mechanism allows us to make an important distinction between cool and hot EF; cEF involves brain regions such as the lateral prefrontal cortex, the parietal cortex, and the anterior cingulate cortex, whereas hEF involves the orbitofrontal cortex and the ventromedial prefrontal cortex [17]. This distinction is supported by patient studies (cEF [62] and hEF tasks [63]). Moreover, neuroimaging research using fMRI has revealed that healthy adults exhibited activation in the lateral prefrontal and parietal regions during cEF tasks [64,65,66] and the activation in the orbitofrontal cortex and ventromedial prefrontal cortex during hEF tasks [67,68].

It should be noted that the lateral prefrontal regions, core regions of cEF, are activated even during hEF tasks [67,69]. For example, Hare et al. [69] asked adults (self-reported dieters) to make decisions about which foods to eat (i.e., a neutral food vs. a tasty but unhealthy food); their neural activities during the decision-making were assessed using fMRI. The results revealed that activity in the ventromedial prefrontal regions was correlated with values related to eating food. Activity in the dorsolateral prefrontal regions increased when the participants exercised self-control (that is, when they declined Liked-Unhealthy items or chose Disliked-Healthy ones) compared to when they failed to exercise self-control. Moreover, there were negative correlations between activation in the left dorsolateral prefrontal regions and the ventromedial prefrontal regions in participants who successfully exercised self-control.

Although fMRI is not easily applied to young children, functional near-infrared spectroscopy (fNIRS) research has evidenced lateral prefrontal cortex activity in preschool children performing cEF tasks [70,71,72]. Moriguchi and Hiraki [71] used fNIRS to investigate the neural correlates for the DCCS task in young children. In this study, 5-year-old children and adults correctly performed the DCCS task and showed significant activation in their lateral prefrontal areas during the task compared to the control (participants sorted blank cards). Meanwhile, 3-year-old children who performed the DCCS task correctly exhibited significant activation in their right lateral prefrontal regions, while those who failed during the postswitch phase exhibited no significant prefrontal activation. These results have been replicated by subsequent studies [73,74].

Research has shown that children can activate their prefrontal regions and other areas, including the parietal regions, during cEF task performance (e.g., DCCS). Indeed, Buss and Spencer [75] reported that activations in the parietal regions could differ between children who performed better during the DCCS task and those who performed worse. A functional link may or may not exist between the prefrontal and the parietal regions during early childhood, but it is thought that such a link may be present at least during middle childhood. Using fMRI, Morton et al. [76] found that the superior parietal cortex and the inferior frontal junction as well as the lateral prefrontal regions can be significantly activated in school-aged children as well as adults. Moreover, a functional connectivity between the lateral prefrontal regions and the inferior parietal cortex, as well as with subcortical regions, has been demonstrated in both adults and school-aged children [77].

**Figure 1:**
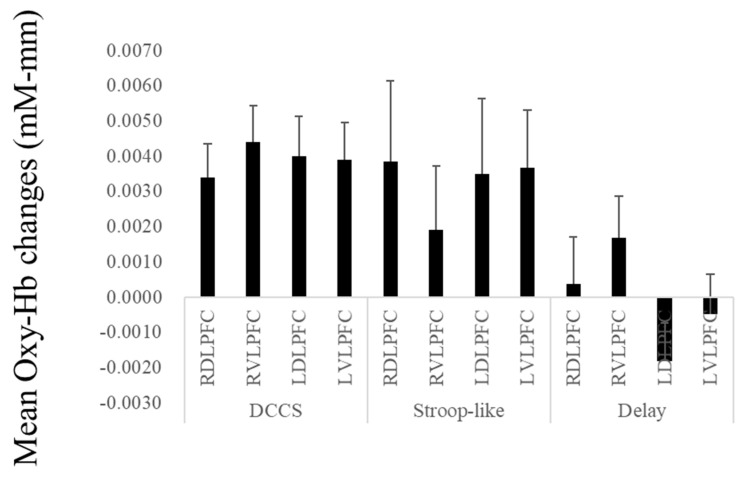
The mean changes in oxy-Hb during the DCCS, Stroop-like, and delay-of-gratification tasks. Error bars indicate standard error. Footnote: RDLPFC, right dorsolateral prefrontal cortex; RVLPFC, right ventrolateral prefrontal cortex; LDLPFC, left dorsolateral prefrontal cortex; LVLPFC, left ventrolateral prefrontal cortex.

Some studies have examined the neural basis for young children’s performance on Stroop-like tasks. Recently, Moriguchi [78] had children complete a task similar to a grass/snow task. In the congruent condition, the children were asked to select cards in a manner that was consistent with the experimenter’s instruction (e.g., the children had to point to a black card when the experimenter said “black”). In the incongruent condition, the children were asked to select cards in a manner that was inconsistent with the experimenter’s instruction (e.g., the children selected a yellow card when the experimenter said “green”). The children performed worse in the incongruent condition compared to the congruent condition. At the neural level, there was significant activity in the children’s lateral prefrontal regions during the task. In this study, the children completed the DCCS task as well as Stroop-like tasks, and no significant differences in terms of lateral prefrontal activation were found between the different tasks (Figure 1).

However, little is known about the neural correlates of hEF in young children. This situation may be attributable to the fact that fNIRS is unable to measure brain activities in deep regions (e.g., orbitofrontal cortex and the ventromedial prefrontal cortex regions) because near-infrared light has limited penetrability [79].

As noted above, adult participants have been known to activate the lateral prefrontal regions during delay-of-gratification tasks. To see if that applied, children were given a delay-of-gratification test and activations in the lateral prefrontal areas of the children during the tasks were measured [80]. Children were prompted to choose a box marked “Now” if they wanted a sticker immediately and “Later” if they wanted to wait until later to obtain more stickers. The findings showed that the lateral prefrontal areas of the children were activated during the tasks; however, the activation appeared to be less pronounced than in cEF tasks. Indeed, children engaged in more activity during the DCCS task compared to the delay-of-gratification task [78] (Figure 1). Importantly, the study also revealed that the DCCS and delay-of-gratification tasks did not significantly correlate with lateral prefrontal activations.

Moriguchi and Shinohara [81] observed activity in the lateral prefrontal regions while young children performed “less-is-more” tasks. As noted above, this type of task involves both cool and hot EF; therefore, it was expected that the children would show lateral prefrontal activations during the task. The results were consistent with this prediction as the preschool children’s prefrontal regions showed activity during the tasks.

Similar to this, other research has shown, even though they did not use hEF tasks, that lateral prefrontal regions may be activated in executive control processes including reward processing [82]. Preschoolers were given a go/no-go task, where researchers contrasted the activations in the ventrolateral prefrontal regions during a control condition (without incentives) with those during conditions with both social (parental face) and non-social rewards (stickers). The findings confirmed that children in the social reward condition were more likely than those in the control condition to exhibit activation in their lateral prefrontal areas. The lateral prefrontal cortex was observed to activate in children in response to social incentives.

In summary, the lateral prefrontal regions may be activated during hEF tasks as well as cEF tasks among both young children and adults. It is unclear whether the core regions involving hEF, such as the orbitofrontal cortex and the ventromedial prefrontal cortex, were reported as activated during cool and hot EF tasks in young children because of technical issues. Nevertheless, previous studies have suggested that the cool-hot distinction may be unclear even with regard to children’s brains.

## 6. Conclusions and Future Direction

Using conceptual distinction, factor analysis, cognitive complexity, and neuroimaging research, we reviewed the cool–hot distinction theoretical EF framework during childhood. First, conceptual analyses suggest that, although cEF is generally distinguishable from hEF, some tasks may include both aspects of EF. Second, research involving confirmatory factor analyses has suggested that both cool and hot EFs can be loaded as different factors; however, some studies have reported that one general factor is sufficient for explaining children’s performance on cool and hot EF tasks. Third, we used the CCC and RC theories to analyze the cognitive complexity of EF and suggested that there is a consistent level of cognitive complexity within cEF and that the cognitive complexity of cEF is similar to that of hEF. Finally, neuroimaging studies suggest that the lateral prefrontal regions are activated both in cool and hot EF tasks; however, it is unclear whether the orbitofrontal cortex and the ventromedial prefrontal cortex are activated during cool and hot EF tasks.

Taken together, our analyses suggest it is reasonable to conclude that cEF may be distinguishable from hEF during early childhood, but this distinction may not always be consistent and clear. This conclusion aligns with previous research proposals that there may be some theoretical and empirical issues in the cool–hot EF framework [21].

Overall, we have three suggestions for future research in this area. First, more research is needed to examine hEF. Conceptually, affective conditions in hEF include not only reward motivation but also positive emotion [33]. The differences in affective conditions were not clearly considered in developmental studies, but recent adult studies suggested that the influence of reward motivation and positive emotion on EF can be partially dissociable [83]. The conceptual analyses and empirical data were needed to clearly define hEF. In terms of assessment, several tasks assess cEF (e.g., Stroop-like tasks, DCCS task), but few tasks have been developed to assess hEF. Indeed, although delay-of-gratification tasks and gambling tasks are widely used, some researchers include a tower task [43], while some include a questionnaire [45] for hEF. More tasks to assess hEF are needed in future research. Moreover, the neural basis for hEF during early childhood is still unknown, though it is important to the distinction between cEF and hEF. As noted above, because of technical hurdles, it is difficult to assess the activation in the core regions involving hEF, such as the orbitofrontal cortex and the ventromedial prefrontal cortex, using fNIRS. Recently, some researchers used fMRI to assess brain activities in young children [84], and we propose that the neural basis of hEF can be clarified directly using such techniques. Alternatively, indirect measures related to reward processing using other methods, such as EEG and gaze pattern, can be useful to assess the neural basis for hEF [85].

Second, the distinction between “cool” EF and “hot” EF may be more graded than categorical. Thus, researchers should consider the extent to which a given task prompts the cool and hot aspects of EF. In other words, the “temperature” of a given EF task (e.g., increasing or decreasing reward salience) [21] and the modulation of approach–avoidance reactions should be considered [28].

Several tasks used in child development studies—not only EF tasks but also other tasks as well—may include both the cool and hot aspects of EF. For example, a sharing task, which is often used in prosocial development research, can include both the cool and hot EF aspects. In this type of task, children are often allocated tokens that can be exchanged with strangers in return for attractive stickers [86]. They are provided with four tokens and asked to choose from three options as follows: (1) keep two tokens for themselves and give two tokens to a stranger, (2) keep three tokens for themselves and give one to a stranger, or (3) keep all four tokens for themselves and give no tokens to a stranger. The children tend to preserve their own resources. In this task, hEF is required to regulate their impulse to gather tokens, and cEF is required to inhibit their natural tendency to choose the third option—that is, keeping four tokens for themselves and giving no tokens to a stranger—in accordance with altruistic sharing (e.g., a two-two sharing of tokens); thus, this task is similar to the “less-is-more” task. The results for this task have revealed dorsolateral prefrontal activation in children, which was related to cEF, during the two-two distribution of tokens, but not during the three-one or four-zero distribution.

Finally, we recommend that future work should be carried out in non-WEIRD (Western, Educated, Industrial, Rich, and Democratic) countries. The importance of diversity in developmental research practice has come into focus, but most research is still biased toward participants from WEIRD backgrounds, and such populations are not representative of all humans [87,88]. Regarding EF, several recent studies have examined the development of EF in non-WEIRD countries, but whether and how “cool” and “hot” EF are distinguished in such countries are still unknown. To provide answers, researchers should examine whether the cool–hot framework of EF can be observed across countries.

To conclude, the cool–hot framework is useful for explaining children’s behaviors, but it is not a decisive assessment tool. In future research, rather than simply classifying EF tasks as cool or hot EF, we recommend describing a given EF task in terms of both cool aspects and hot aspects.

## Figures and Tables

**Table 1:** Classification of cool and hot EF tasks.

Cool	Cool and Hot	Hot
Standard DCCS Stroop-like Grass/snow Luria’s hand game	Emotional DCCS Less-is-more	Gambling task Delay-of-gratification

**Table 2:** Cognitive complexity of cool and hot EF tasks.

	CCC Theory		RC Theory	
Cool				
DCCS	Higher-order rules		Ternary	
Stroop	Higher-order rules		Conditionally, ternary	
Hot				
Gambling	Higher-order rules		Ternary	
Delay-of-gratification	Higher-order rules?		Ternary	

## Data Availability

Not applicable.
